# Extensive use of Fish Aggregating Devices together with environmental change influenced the spatial distribution of a tropical affinity fish

**DOI:** 10.1038/s41598-019-41421-9

**Published:** 2019-03-20

**Authors:** Mauro Sinopoli, Valentina Lauria, Germana Garofalo, Teresa Maggio, Tiziana Cillari

**Affiliations:** 1 Stazione Zoologica Anton Dohrn Napoli, Palermo branch, Lungomare Cristoforo Colombo n. 4521 (ex complesso Roosevelt) Località Addaura, 90149 Palermo, Italy; 2 IRBIM - Istituto per le Risorse Biologiche e le Biotecnologie Marine, CNR - National Research Council, Via Luigi Vaccara 61, 91026 Mazara del Vallo (TP), Italy; 3 Institute for Environmental Protection and Research (ISPRA), BIO-CIT. Lungomare Cristoforo Colombo n. 4521 (ex complesso Roosevelt) Località Addaura, 90149 Palermo, Italy

## Abstract

Since the early 1990s of last century the spread of *Caranx crysos* a thermophilic fish species in the Mediterranean Sea has increased due to sea warming. Simultaneously, a large and unregulated use of fish aggregating devices has been recorded, and these devices seem to influence fish spatial distributions. Here we used a multidisciplinary approach to investigate the influence of environmental change and FAD presence on the spatial distribution of the tropical affinity fish species *Caranx crysos* across the Mediterranean Sea. Data suggested that the presence of *C*. *crysos* has increased progressively since 1990 towards the northwest side of the Mediterranean Sea, with the greatest number of recent findings occurring in zones with higher densities of FADs. The spatial distribution models show how the extensive use of FADs in combination with changes in environmental conditions may have indirectly facilitated the spread of the thermophillic *C*. *crysos* population across the Mediterranean Sea.

## Introduction

Floating objects affect the distribution of both juveniles and adults of some pelagic fish species. High fish densities have been reported around drifting algae and man-made objects such as rafts, i.e. fish aggregating devices (FADs)^1^. Fishermen have been placing FADs to attract and/or aggregate fishes for more than 1,000 years. Fish aggregating devices are applied world-wide with significant increases in the yields of industrial, artisanal and recreational fisheries^2^.

While in the world’s oceans’ FAD fisheries have grown at the industrial level with the introduction of drifting FADs^2^, in the Mediterranean this practice has remained active for centuries using anchored FADs. In fact, these are still in use in the western and central Mediterranean Sea by artisanal fisheries^3^.

Because of the widespread use of FADs in the marine environment, their effects on the associated fish fauna in terms of composition, feeding habits and temporal trends of juvenile settlement have been studied^4–7^. Some studies have suggested that FADs might modify the feeding regime of some fish species with poor growth conditions and survival rates (e.g. *Coryphaena hyppurus* and some Tuna-like fish species). In addition, FADs seem to have a negative effect on fish distribution because of trap-effects that disrupt migratory patterns^2^. Although FAD effects in the marine environment have been studied, knowledge on the role of FADs as a tool to facilitate spatial distribution of marine species is very scarce.

In the Mediterranean Sea, FAD fisheries are very important and mainly target common dolphinfish (*Coryphaena hippurus* Linnaeus, 1758) and different species as by-catch (e.g. greater amberjack *Seriola dumerilii* Risso, 1810 and bluefin tuna *Thunnus thynnus* Linnaeus, 1758^5–7^). This activity is common in southern Italy, Tunisia, Malta and Spain where it has historical roots in the Balearic Islands^3^. In all of these areas the fishing areas, the materials with which FADs are made and fishing techniques are very similar^3^.

In Sicily (central Mediterranean), traditional FADs called “*cannizzi*” are usually set at sea by fishers in late summer and they start to fish the target species (*C*. *hippurus*) from September to January. The FADs are made of palm fronds joined to floating objects (plastic bottles or styrofoam bodies) and are anchored to the bottom with polypropylene rope and large stones or concrete blocks. At the beginning of the 20th century this practice used few boats with few FADs, but quickly developed with the advent of motorized boats and the increasing demand for fish. According to the latest censuses carried out in Sicilian seas in 1996, 19,000 FADs are used every year^3^. Nowadays, these systems are arranged in lines as far as 20 NM from the coast made up of numerous series of FADs that are spaced about 1 km from each other and are settled on the sea bottom deeper than 1,500 m. Due to their deep anchoring depth, FADs are subjected to a wide fluctuation that makes it difficult to find them. For this reason, fishers prefer lines of FADs that represent shipping tracks and facilitate their recognition^8^.

The fish fauna associated with FADs in the Mediterranean Sea is made up of eleven species. In general, it consists of juveniles of species whose adults are pelagic, reef associated or nectobenthonic species^4,5,9^. Among these the blue runner (*Caranx crysos*, Mitchill, 1815) is one of the main species normally associated with FADs from September–January. This fish species feeds on zooplankton from about 2 cm to about 16 cm total length^10^ and leaves the FADs when it is able to swim in school^5,11^. Sub-adults and adults of *C*. *crysos* prefer littoral waters where they live until they reach a maximum total length of 70 cm^12^.

The blue runner is distributed in the Eastern Atlantic from Senegal to Angola and in the Western Atlantic from Nova Scotia to Brazil. This species is thermophilic because it is mainly distributed in the southeastern Mediterranean basin, although it has also been caught in the southern part of the western basin^13^. The abundance and spatial distribution of the blue runner in the Mediterranean Sea are increasing over time, and this species is regularly caught in Tunisian waters, near Lampedusa Island and Sicily and in the central**-**southern Tyrrhenian Sea up to the Gulf of Naples^14,15^. Despite its presence being well established in the Mediterranean Sea, not much is known about which factors affect its spatial distribution. A first attempt to reconstruct the northward spread of the blue runner in the Mediterranean Sea was made by collecting all of the historical records present in bibliography, museum and field data^14^. This study suggested a regression of the distribution of this species until the 1970s and an important increase of northern spread since the 1990s. The expansion of its spatial distribution because of water warming was similar to many other southern marine species that along the last century moved northward as a consequence of changing in the environmental conditions (such as increase of water temperature)^16^. Many of the new finds of *C*. *crysos* are reported in areas with a high presence of FADs^15^. In addition, some studies have highlighted the positive relationship between the presence of FADs and the survival rates of small juvenile *C*. *crysos* in the central Mediterranean Sea. This is because FADs act as a refuge from predator attacks and allow fish species to escape from fishing gear^7,17^. Moreover, depending on their distance from the coast, FADs seem to also facilitate the settlement of other juvenile fish species in the coastal zone (e.g. Greater amberjack, *Seriola dumerili* Risso 1810^18^). These observations led us to hypothesize that the presence of FADs in concomitance with the changes in environmental conditions in the Mediterranean Sea could indirectly influence the northward spread of *C*. *crysos*.

The aim of this study was to assess which factors influence the spatial distribution of *C*. *crysos* in the Mediterranean Sea through two complementary approaches:

1. The reconstruction of historical records of *C*. *crysos* and a spatial modelling approach to study the relationships among its presence, environmental factors (i.e. sea surface temperature, sea surface salinity and chlorophyll concentration) and the presence of FADs.
2. A field experiment to understand if the presence of FAD systems favours *C*. *crysos* in its dynamic approach to the coast. Specifically, we hypothesized that offshore FADs attract the first fish settlers and the FAD systems deployed from off-shore to in-shore act as stepping stones, driving them towards the coast where reefs are located. In this case, mean abundance and size should decrease over time in offshore areas and increase in inshore areas.

## Methods

### Data collection

We used the 77 records of presence of *C*. *crysos* in Mediterranean reported in the database of Psomadakis *et al*.^14^ that were based on literature searches, including investigations of systematic collections, registers and databases of the main Italian and foreign museums. We updated this database with recent literature available on the principal scientific search sources (Scopus, Web of science, Scholar), including all Mediterranean records of the presence of *C*. *crysos* that indicated the area and geographical coordinates where the species was caught, recorded or observed.

To visualize the expansion progress of *C*. *crysos* in the Mediterranean Sea, point data (sites where the species has been recorded/captured/observed) were imported into ArcGIS 10.3 ESRI and geographically represented as cumulative occurrences for the following four periods: from the year of first record (1817) until 1990, 2000, 2010 and 2017.

Data on presence, abundance and distribution of FADs in the Mediterranean Sea come from a review^3^. More recent information on the distribution of FADs is not present in the literature, however the current distribution and number of FADs is similar to that described in 2001. In other regions of the Mediterranean Sea (e.g. Spain, Malta, Tunisia and Greece) the number of FADs seems not to have changed in recent years (Morales-Nin, Gatt, Besbes and Vassilopoulou personal comments). In fact, according to a direct request to researchers from Spain, Malta, Tunisia and Greece (as well as our knowledge of Italy) there have not been large variations in the number of FADs (Morales-Nin, Gatt, Besbes and Vassilopoulou personal communication).

The presence, distribution and number of FADs were mapped together with the information about the areas of influence of FADs (as polygons), differentiated for total number of FADs relative to the periods and typology of deployment at sea. Four FAD deployment typologies were present:

1. near line: lines of several FADs placed perpendicular to the coast starting from a close distance (0.5 NM) up to about 20 NM;
2. far line: lines of several FADs placed perpendicular to the coast starting from a distance of about 6 NM up to 20 NM;
3. random FAD: several FADs placed randomly at sea without forming lines, without a definite distance from each other and a definite distance from the coast (reaching distant areas up to 40 NM from the coast);
4. mixed: a mix of “far-line” and “random FAD” typologies (even up to 20 NM away).

We decided to perform the successive analysis considering two time periods, before 1990 and after 1990. This year was chosen because it corresponds to the beginning phase of *C*. *crysos* expanding north in the Mediterranean Sea^14^ as well as the increase in the number of FADs employed in the southern Tyrrhenian Sea^3^.

Sea surface temperature (SST), surface salinity (using the Practical Salinity Scale) and chlorophyll concentration were selected as environmental predictors of *C*. *crysos* distribution because surface salinity and SST are strongly related to marine system productivity as they can affect nutrient availability, metabolic rates and water stratification^19,20^. It has been shown that these environmental factors influence the distribution patterns of demersal species^21,22^. Chlorophyll concentration can be used as a proxy of primary productivity in marine systems and provides indications of areas of high fish abundance^23^, particularly juvenile stages (probably because of higher food availability).

Monthly mean of the environmental variables (of continuous type and at the Mediterranean scale) were obtained from the online catalogue E.U. Copernicus Marine Service Information (http://marine.copernicus.eu) for the period from 1955–2015 for SST and surface salinity, while chlorophyll data are relative to the period from 1999–2015. All digital continuous maps have a spatial resolution of 0.063 degree × 0.063 degrees^24^.

These data were processed in ArcGIS 10.3 and the mean of each environmental variable and its associated variability was calculated for the two time periods (before and after 1990). The variables we tested were SST and sea surface salinity (SST, standard deviation of SST, sea surface salinity and standard deviation of sea surface salinity) and, only for the period after 1990, chlorophyll and the standard deviation of chlorophyll.

### Modelling approach

We used a species distribution model (SDM) to study the distribution of *C*. *crysos* in the Mediterranean Sea in relation to the environment and FAD presence/type. The set of explanatory variables used to construct the model is shown in Table 1.

**Table 1:** Explanatory variables used in the MaxEnt model.

Variables	Reference period	Labels	Variables typology
Monthly mean sea surface temperature (°C)	1955–1990	sst_B90	continuous
	1990–2015	sst_A90	
Standard deviation of sea surface temperature (°C)	1955–1990	std_sst_B90	continuous
	1990–2015	std_sst_A90	
Monthly mean salinity (PSU)	1955–1990	sal_B90	continuous
	1990–2015	sal_A90	
Standard deviation of salinity (PSU)	1955–1990	std_sal_B90	continuous
	1990–2015	std_sal_A90	
Chlorophyll (mg/m^3^)	1999–2015	chl_p99	continuous
Standard deviation of chlorophyll (mg/m^3^)	1999–2015	std_chl_p99	continuous
Total number of FADs	50s-1990	fad_B90	categorical
	1990–2017	fad_A90	
FADs typology	50s-1990	type_B90	categorical
	1990–2017	type_A90	

Two models relative to the periods before and after 1990 (named B90 for before 1990 and A90 for after 1990) were built based on the occurrences of *C*. *crysos* coupled with maps of environmental data and FAD distribution. The SDMs were built using the maximum entropy method (software MaxEnt version 3.4.1^25^, available at http://biodiversityinformatics.amnh.org/open_source/maxent) which only uses presence data and a user-defined number of randomly selected points (pseudo-absences) and combines these with the covariates to construct a probability of presence map (for each cell of the map the probability of presence ranges from 0–1). We chose a value of 0.5 as a threshold to differentiate low and high probability of presence^26,27^. Models were tested for sensitivity using the receiver operating characteristic (ROC) curve and we assessed the area under the receiver operating characteristic curve (AUC^26^). An AUC value of 0.5 indicates that the model performs no better than a random model, whereas a value of 1 indicates that the model is fully capable of distinguishing between occupied and unoccupied sites. AUC values of 0.7 and 0.9 indicate very good discrimination while values >0.9 indicate excellent discrimination.

For each of the two models (B90 and A90) MaxEnt started with a uniform distribution of occurrence probability for *C*. *crysos* over the entire Mediterranean basin and conducted an optimization routine that iteratively improved the model fit, measured as the loss of entropy (i.e. the “gain” of information). MaxEnt also provides a jackknife procedure that assesses which of the variables have the greatest influence on the prediction by calculating the per cent contribution, which is the contribution of a predictor variable that fits best with the model in each iteration of the training algorithm. This measure is related to the path that the MaxEnt algorithm uses to increase the gain of the model. MaxEnt also provides the permutation importance, which is calculated by random permutation of the values of a variable among the training presence and background data and measuring the resultant decrease in training AUC following the recalculation of the model. Per cent contribution and permutation importance are normalized and expressed as a percentage. Finally, jackknife also provides a leave-one-out test of variables’ importance in which the model is calculated with only one variable and then without this using all the other remaining variables. This procedure was tested for the training and test data as well as for the AUC of the test data.

### Field experiment: FADs’ influence on C. crysos settlement towards the coast

**Figure 1:**
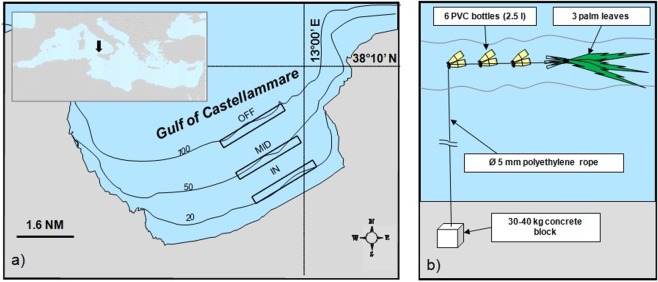
(**a**) Study area for the experimental session. (**b**) Construction scheme of FADs (fish aggregation devices) used by fishermen and for experimental fields.

The experimental study was performed in 2014 in the Gulf of Castellammare (38°03′N, 12°54′E) to study the settlement of *C*. *crysos* and the two others species *Trachurus trachurus* and *Balistes capriscus*. An experimental FAD systems was deployed from April to December, period covering the recruitment of the three selected species^5^. This area is located on the northwest coast of Sicily (Fig. 1a) and its coastline is over 70 km long and covers an area of about 300 km^2^. In March 2014 three experimental FAD systems were deployed and managed up to the end of the study (December 2014) in three areas: inshore (IN) (0.9 NM from the coast; 20–25 m deep), mid-shore (MID) (1.4 NM from the coast; 50–55 m deep) and offshore (OFF) (2.8 NM from the coast; 100–110 m deep) (Fig. 1a). Each system included 20 palm leaf FADs (Fig. 1b) set at 500 m from each other. During the whole study period professional and recreational fishing activities were banned within the experimental area.

In three different time periods (T1, T2 and T3) underwater visual censuses were carried out on three randomly selected FADs^11^. Together with *C*. *crysos*, other juvenile fish species (*B*. *capriscus* Gmelin, 1789 and *T*. *trachurus* Linneaus, 1758) were also sampled. This allowed a comparison of the mechanism of use of FADs between a species that becomes demersal after the period of association with FADs (*B*. *capriscus*) and one that remains coastal pelagic (*T*. *trachurus*). In detail, sampling of *C*. *crysos* and *B*. *capriscus* was performed on June 28 (T1), September 5 (T2) and November 14 (T3), while sampling of *T*. *trachurus* was carried out on May 17 (T1), June 12 (T2) and July 18 (T3).

A single SCUBA diver performed all censuses (MS). The diver approached the FADs down current from a distance of about 20 m. All fish species were counted at each FAD system separately for three classes of total length (TL): small (SM, up to 10 cm), medium (ME, 11–14 cm) and large (LA, >14 cm).

For each species the data were processed by analysis of variance (ANOVA) in which the three fixed orthogonal factors were distance from the coast (DIST) with three levels (OFF, MID and IN), size classes (SIZE) with three levels (small, medium and large) and period (PE) with three levels (T1, T2 and T3)^28^. Homogeneity of variances was checked with Cochran’s C tests. Student-Newman-Keuls (SNK) tests were used to separate the mean (at α = 0.05) if significant differences in the ANOVA were found^28^. The GMAV 5.0 software (University of Sidney) was used to perform all of the statistics.

## Results

### Records of  C. crysos, environmental factors and FAD number

**Figure 2:**
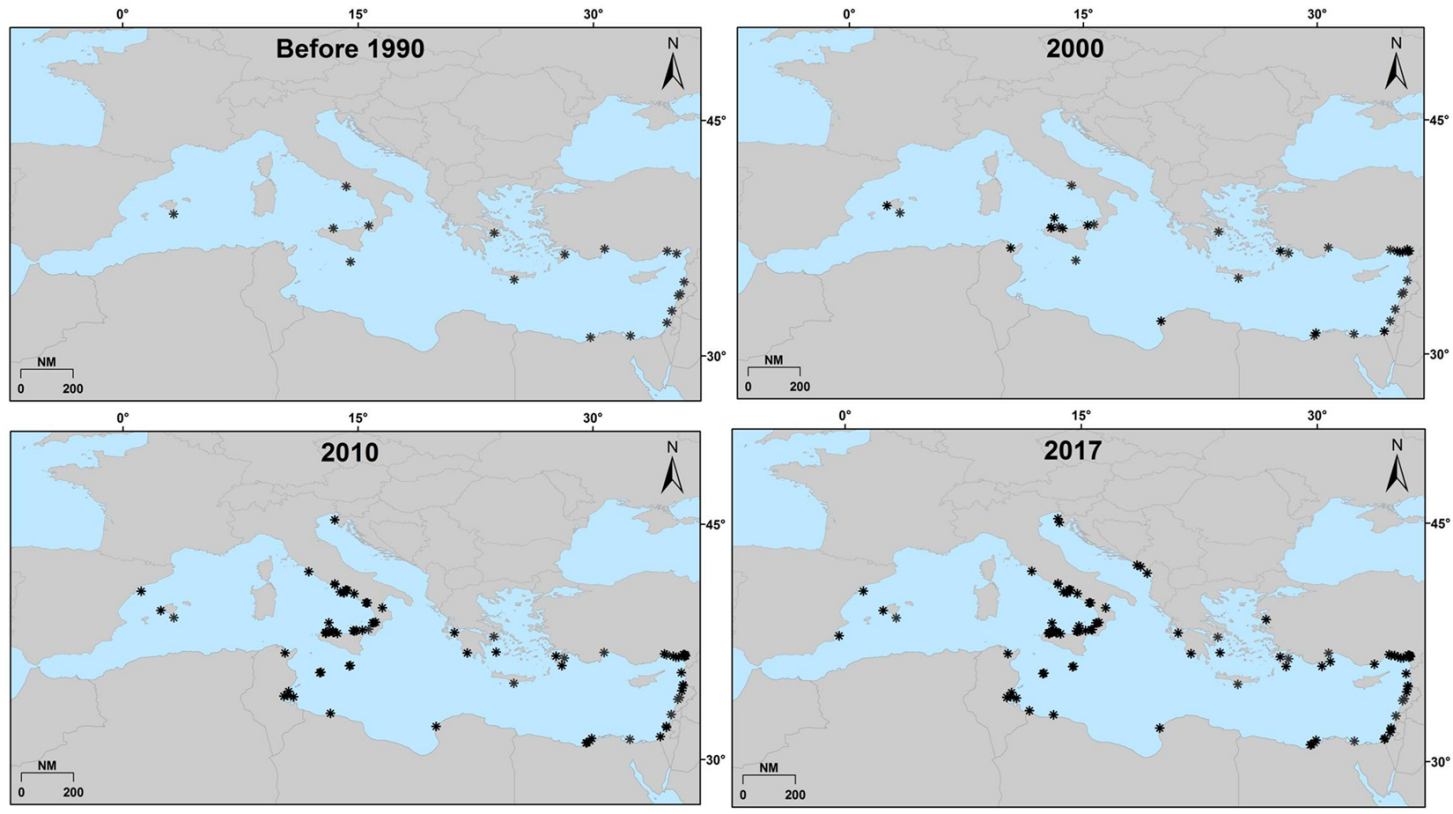
Cumulative occurrences of *C*. *crysos* (circles indicate records) in the Mediterranean Sea from 1817 to October 2017 for four periods (107 total records).

The database of Psomadakis^14^ was updated to October 2017 with a total of 112 records (see Appendix S1 for details) of *C*. *crysos* in the Mediterranean Sea with the oldest record dating back to 1817. Before 1990 the group of records is from the southeast Mediterranean (14 of 22 total records; Fig. 2). Over time, the number of records has increased progressively to the northwest side of the Mediterranean Sea. As early as 2000, the greatest number of new finds occurred in the southern Tyrrhenian Sea (51 of 90 total records; Fig. 2).

**Figure 3:**
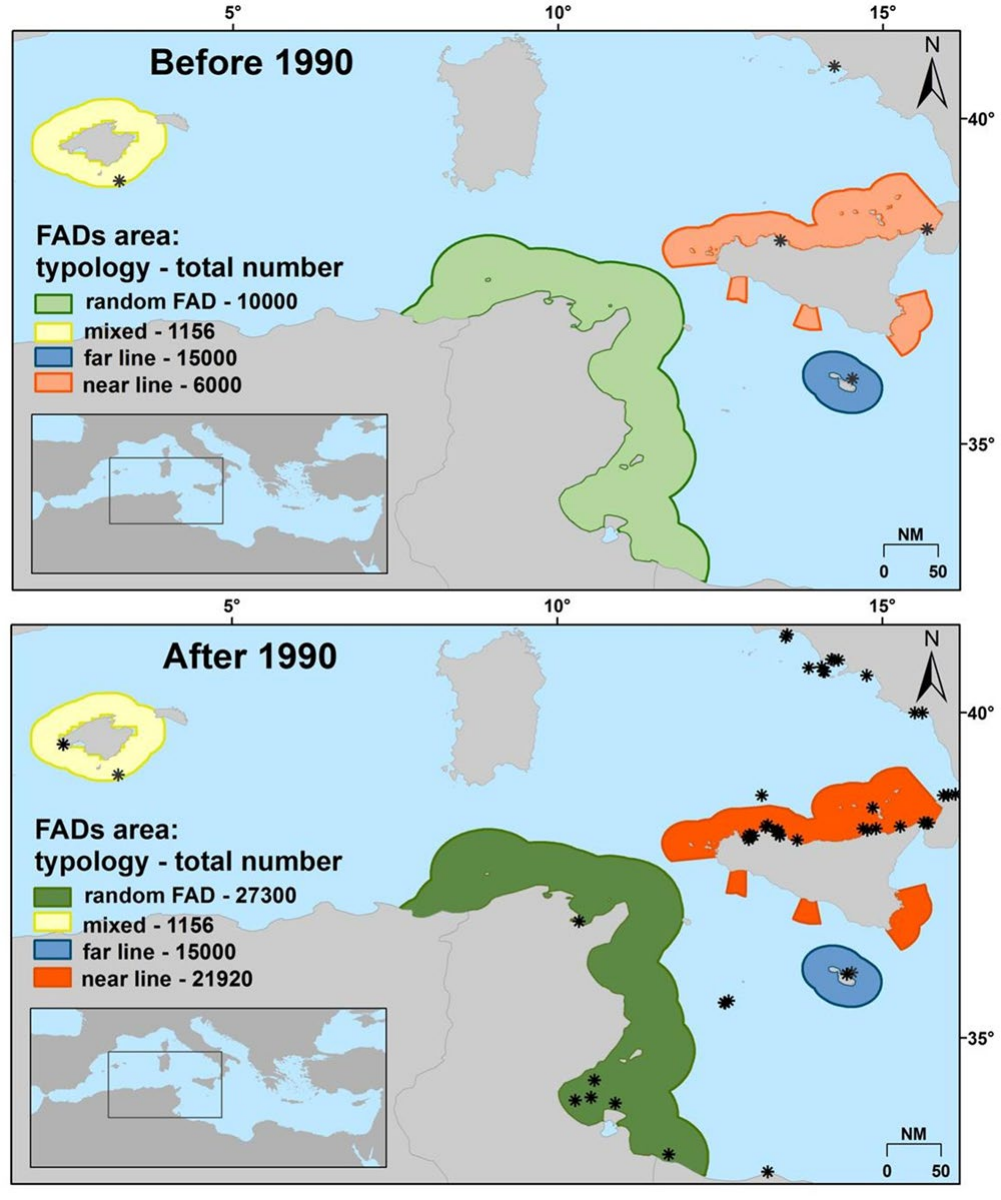
Temporal trends of total number and deployment typology of FADs over the two time periods in the southern Tyrrhenian Sea and western Mediterranean Sea with indication of *C*. *crysos* records (asterisks indicate records).

Based on the re-elaboration of data from Morales-Nin *et al*.^3^, 6,000 near line FADs were deployed every fishing season before 1990 in the southern Tyrrhenian Sea, while 10,000 random FAD typology FADs were placed in Tunisian waters. Between 1990 and 2001, the number of FADs increased to 21,920 in the southern Tyrrhenian Sea and 27,300 in Tunisian waters (Fig. 3). In the western Mediterranean the only FADs present over all the periods were the 1,156 of the Balearic Islands that are placed in a mixed way (Fig. 3). Also, in Malta the total number of FADs remained fixed over time with 15,000 far line FADs deployed (Fig. 3). Currently, the total number of FADs and their deployment typology are equal to those of 2001. The maps of the six environmental predictors used for model construction relative to the two time periods (before and after 1990), with the exception of chlorophyll for which data are not available before 1990, are reported in Fig. S2 (see Appendix S2).

### MaxEnt species distribution models

#### Before 1990 model (B90)

**Figure 4:**
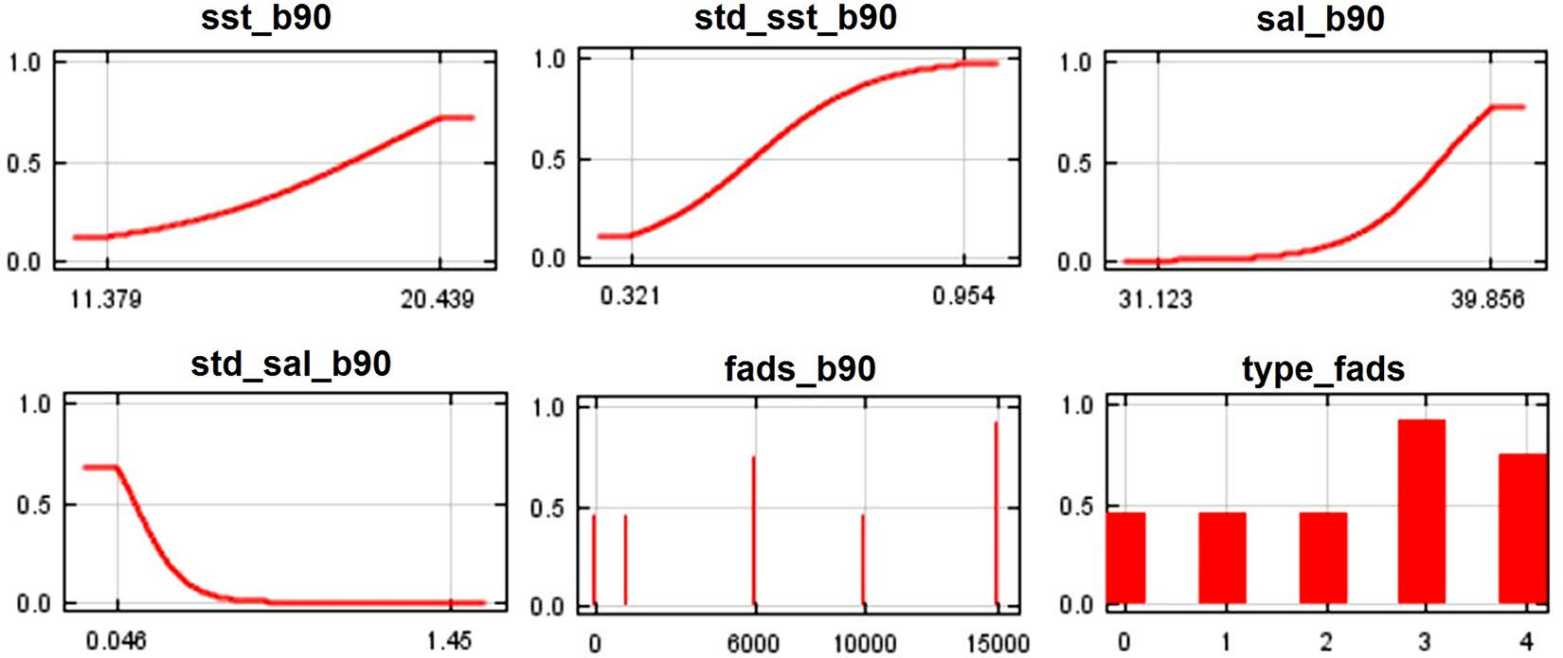
Partial dependence curves showing the marginal response (i.e. for constant values of the other variables) of *C*. *crysos* in the Mediterranean Sea to the six explanatory variables of the B90 model.

The AUC scores for training and testing data were 0.828, suggesting that the models had a very good discriminating ability. SST contributes 28.5% to the B90 model (Table 2). Despite its consistent contribution, this variable showed less importance when assessed with the other jackknife tests (per cent permutation importance, training gain without, training gain with only; see Table 2). The higher probability of *C*. *crysos* presence is in areas where SST reaches 20.4 °C (Fig. 4). The standard deviation of SST in the B90 model showed high values of per cent contribution and permutation importance (33.1% and 60.1%, respectively) (Table 2). This variable decreases the gain more when it is omitted, and therefore appears to have the most information that is not present in the other variables (Table 2). In this case the species probability of presence is higher when this variable reaches values between 0.5 °C and 0.9 °C (Fig. 4). The standard deviation of salinity showed values of contribution of 15.8% and permutation importance of 33.8% (Table 2). It has the highest gain when used alone, which therefore appears to have the most useful information by itself (Table 2). For this variable *C*. *crysos* had a higher probability of presence at values of salinity standard deviation near 0.04 (Fig. 4). Total FAD number contributes 18.8% to the B90 model (Table 2), the probability of presence of *C*. *crysos* was associated with coastal areas where the number of FADs was 6,000 and 15,000 (Fig. 4). The variable related to type of FADs showed lower values of all variables of jackknife analyses having an irrelevant role (Table 2). The highest species presence probability occurs for categories 3 and 4 (far and near lines) of FAD deployment (Fig. 4).

**Table 2:** Summary of jackknife test of variable importance for the MaxEnt model of *Caranx crysos* distribution in the Mediterranean Sea.

Variables	SST	Std_sst	Sal	Std_sal	Chl	Std_chl	FADs (num.)	Type
***B90***								
% Variable contribution	**28**.**5**	**33**.**1**	0.0	**15**.**8**	—	—	**18**.**8**	3.7
% Permutation importance	2.8	**60**.**1**	0.0	**33**.**8**	—	—	0.0	3.3
Training gain without	0.50	**0**.**40**	0.53	0.47	—	—	0.53	0.53
Training gain with only	0.15	0.21	0.17	**0**.**21**	—	—	0.10	0.10
***A90***								
% Variable contribution	3.8	0.1	**20**.**6**	**18**.**2**	**29**.**8**	0.8	**26**.**4**	0.3
% Permutation importance	7.2	0.2	9.7	**16**.**7**	**56**.**0**	5.1	5.0	0.2
Training gain without	1.16	1.22	1.09	0.94	**0**.**88**	1.20	1.22	1.22
Training gain with only	0.02	0.00	0.23	0.23	0.22	0.09	**0**.**29**	0.29

SST = sea surface temperature; Std_sst = Standard deviation of sea surface temperature; Sal = Salinity; Std_sal = Standard deviation of salinity; Chl = Chlorophyll; Std_chl = Standard deviation of chlorophyll; FADs (num.) = Total number of FADs; _ = no value available.

#### After 1990 model (A90)

**Figure 5:**
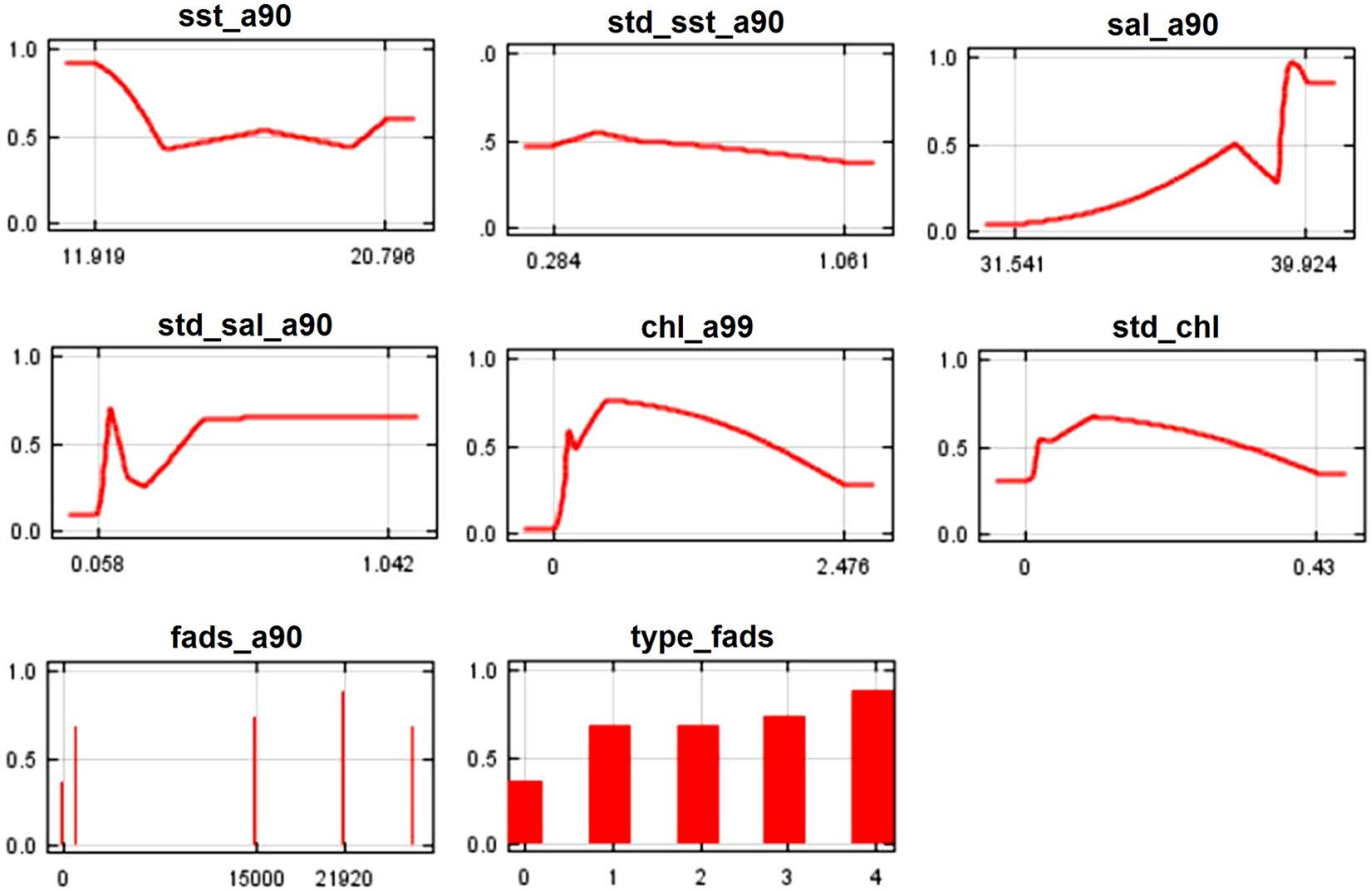
Partial dependence curves showing the marginal response (i.e. for constant values of the other variables) of *C*. *crysos* in the Mediterranean Sea to the eight explanatory variables of the A90 model.

In the A90 model the AUC scores showed a value near 0.893, suggesting that the model had a very good discriminating ability. Salinity contributes 20.6% (Table 2) and showed less importance in the jackknife analyses (Table 2). There is a higher likelihood of finding *C*. *crysos* in areas where salinity reaches values of about 39 (Fig. 5). The standard deviation of salinity showed a contribution of 18.2% and a permutation importance of 16.7% (Table 2). The probability of finding *C*. *crysos* is in correspondence with areas where this variable has a value near to 0.06 or in a range between 0.3 and 1.0 (Fig. 5). Chlorophyll was the explanatory variable with the highest per cent contribution (29.8%) and permutation importance (56%) (with the most information that is not present in the other variables because of the lowest value of training gain without the variable was 0.88; Table 2). The higher presence probability of *C*. *crysos* is in regions where the concentration of chlorophyll is near 0.4 mg Chl a m^−3^ (Fig. 5). Total FAD number contributes 26.0% (Table 2). This variable also showed the highest gain when used in isolation, and therefore appears to have the most useful information by itself (Table 2). The presence probability of *C*. *crysos* is positively associated with areas where the number of FADs was over 10,000 (Fig. 5). Also, in the A90 model the variable related to type of FAD deployment showed a lower value of all variables from the jackknife analyses and thus having an irrelevant role (Table 2). In this case the highest probability of species presence occurs for the categories of FAD deployment from 1–4 (Fig. 5).

#### *Caranx crysos* distribution maps

The two spatial models of the potential distribution of *C*. *crysos* for the two time periods before (B90) and after (A90) 1990 are shown in Fig. S3A,B reported in Appendix S3. Our results suggest that before 1990 (B90 model) the probability of presence of *C*. *crysos* was higher in the eastern Mediterranean and in other areas located on the north coast of Sicily and around Malta. A different distribution pattern is observed after 1990 (A90 model) where there was a longitudinal shift of presence probability from east to west for this species (i.e. higher values are predicted in correspondence of the coastal waters of Egypt and Israel, Tunisian waters, northern coast of Sicily, Tyrrhenian Sea and Balearic Islands).

#### FADs’ influence on C. crysos settlement from offshore to the coast

**Figure 6:**
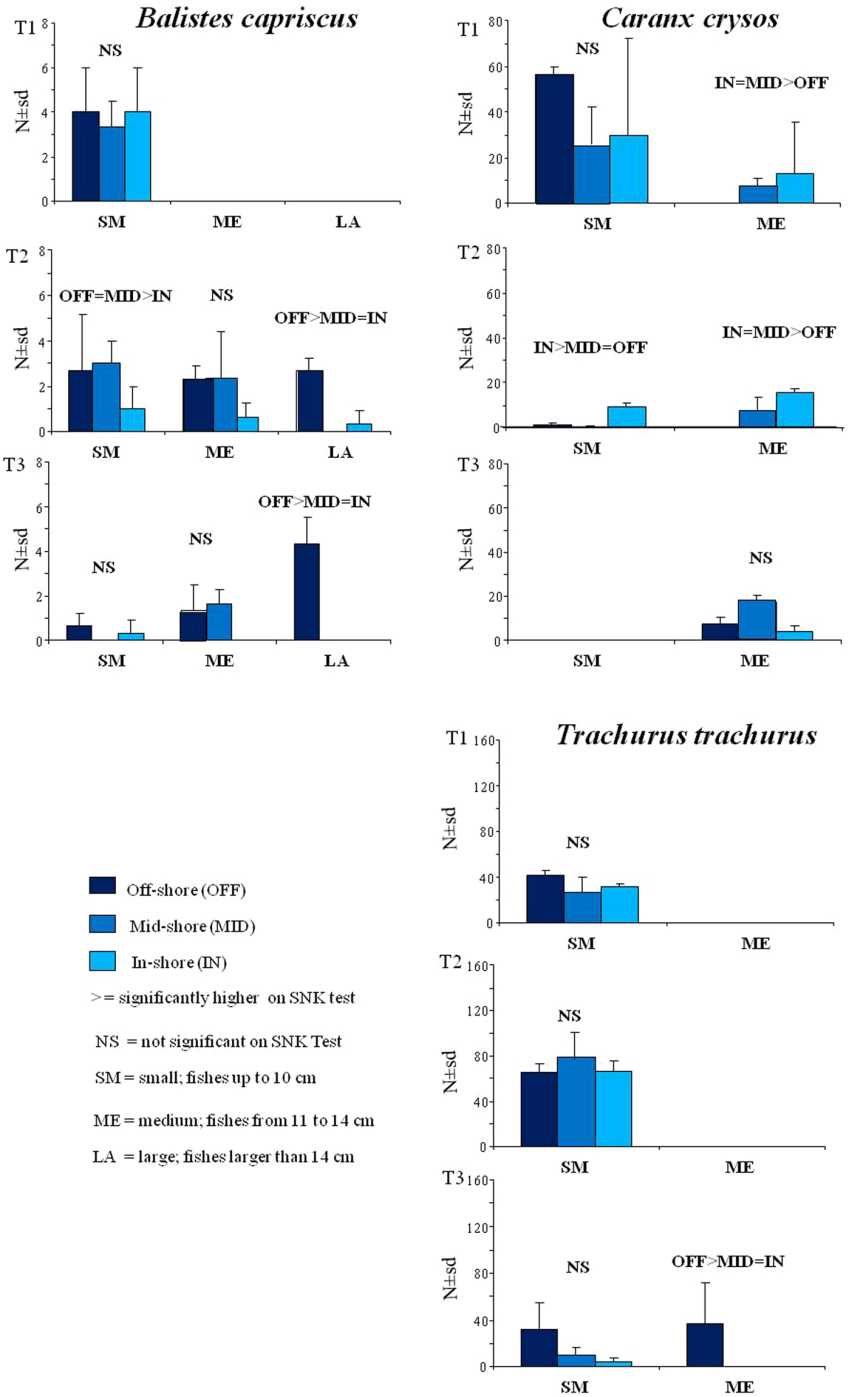
Mean abundance (±standard deviation) of the three fish species related to the three zones (OFF, MID, IN) divided into three periods (T1, T2, T3) and for the three size classes (SM, ME, LA). The figure shows the results of pair comparisons performed by the SNK test.

The results of the three ANOVAs on the three species (*C*. *crysos*, *B*. *capriscus and T*. *trachurus*) showed significant differences (*p* < 0.01) in the interaction between the three factors (Fig. 6). At time T1 no significant differences were found between the distribution of fishes for the three distances from the coast for all species except for the medium size class of *C*. *crysos*, where MID and IN distances showed mean abundances greater than OFF. At time T2 for the small class of *B*. *capriscus*, significantly higher abundances were found in the OFF distances and MID than at IN, while in the large class, abundances at the OFF distance were significantly higher than at MID and IN. The mean abundance of *C*. *crysos* at time T2 was significantly higher in the small class at IN distance than at MID and OFF and in the medium class at IN and MID distances than at OFF. At time T3 in the large classes of *B*. *capriscus* and in the medium class of *T*. *trachurus* the mean abundance was significantly greater at the OFF distance than at MID and IN.

## Discussion

This study shows the influence of FADs presence and environmental conditions on the spatial distribution of *C*. *crysos* in the Mediterranean Sea in two different time periods.

The study of distribution and expansion of marine species based on the analysis of records from literature has been widely used^14,29–33^. However, the use of historical data records can also present limits because the number of records from the literature is not always an indicator of real abundance of a species in a zone. In fact, the number of records can vary according to the historical period due to the development of the research or according to the level of research activity of different countries (for example, a well studied area has more possibilities to get information about certain species). Therefore, we acknowledge that the number of records used in our study might be influenced by the increase of research funds and opportunities in the latest years, but we believe that this has not significantly affected our results because this bias has constantly affected all areas of study in the Mediterranean with the exception of Libya and Algeria^14^. Moreover, two independent models were developed for the periods before and after 1990 and this contributed to mitigating possible biases.

The historical distribution of FADs in the Mediterranean basin was reconstructed using all existing information^3^, and these data along with environmental variables were used to study the spatial distribution of this species during two time periods using a Maxent model.

### Environmental predictors and the role of FADs

Temperature and its variation (standard deviation) were the main predictors of the spatial distribution of *C*. *crysos* in the B90 model. This species showed a preference for temperatures near 20 °C, and notably for areas where the temperature variations were greater. The importance of the standard deviation of SST is strengthened by the fact that it is significant for three indicators of the jackknife analysis (Table 2). Temperature has been used in other studies on the distribution ranges of marine species^29,31,34^. The temperature variable is particularly relevant for non-native marine species, however its effect on species spatial distribution is not always clear. For example, while an increase in temperature contributed to the geographical expansion of the angular crab (*Goneplax rhomboids*) in the North Sea^34^, this variable does not seem to influence habitat suitability for the bluespotted cornetfish (*Fistularia commersonii*) in the Mediterranean Sea^31^. Our results for the B90 model support that a higher sea water temperature contributed to *C*. *crysos* distribution shifts in the Mediterranean Sea, which is similar to other studies on the expansion of *C*. *crysos*^14,35^ and other thermophilic species in the Mediterranean Sea^32,33^.

Salinity has been suggested to influence the habitat suitability of pelagic fish species (e.g. *Trachurus trachurus* and *Trachurus mediterraneus*^36^) as well as demersal fish species (e.g. *Pleuronectes platessa*, *Raja clavata* and *Centrophorus granulosus*^21,37^), however none of the above studies has used the salinity variation as a predictive variable. In our results low values of salinity standard deviation in the A90 model are associated with the probability of presence of *C*. *crysos*. This is different from what has been previously suggested for this species, which can also live in coastal transitional environments such as estuarine and brackish water^38^. We also found that the number of FADs for the period B90 was an important predictor of the distribution of *C*. *crysos*, which was different from our initial hypothesis that FADs would have an influence on the distribution of *C*. *crysos* only after 1990. This could be explained by the fact that in 1990 (about 40 years after the advent of FAD use) these devices could already indirectly start to facilitate the expansion of *C*. *crysos* along the exposed coastal areas.

The A90 model (in which also the influence of chlorophyll was tested, in contrast to the B90 model) revealed a new and different perspective on the variables that regulate the probability of presence of *C*. *crysos* (Table 2). Our results suggest that chlorophyll also plays an important role in the spatial distribution of *C*. *crysos* (Table 2). Primary productivity is a key environmental parameter that has been shown to influence the distribution of marine species^29,34,39^. This variable seems to also influence the expansion of alien species in the Mediterranean Sea^31^. Our results suggest that *C*. *crysos* prefers areas where the salinity reaches 39, which is comparable to other species such as *F*. *commersonii* and the two pelagic fishes *T*. *trachurus* and *T*. *mediterraneus*^31,36^. As for the B90 model, there are no references that use standard deviation of salinity to which we can compare and discuss our results. The number of FADs was also an important variable that influenced the probability of presence of *C*. *crysos* after 1990 (Table 2). This result suggests that even if the two models B90 and A90 are different in terms of the explanatory variables used, the number of FADs played a key role, and the higher presence of FADs was associated with an increase in the population of *C*. *crysos* in the spatial distribution of this species in the Mediterranean Sea.

A similar approach has been used in the past^39^ in a study on the distribution of whale sharks the author applied a habitat suitability model on two different time periods using the same explanatory variables. In his study, Mckinney *et al*.^39^ found a positive relationship between the distance of whale shark record points and artificial objects such as rigs, suggesting that these structures act as hot spot for many reef and pelagic species. In the case of *C*. *crysos*, the positive effect of the presence of FADs on its occurrence throughout the Mediterranean Sea could be thought as an indirect effect whereby years of FAD presence has increased the life success of juveniles with repercussions on coastal adult populations. Finally, it is important to note that the B90 and A90 models represent two different models that cannot be compared. This is due to the presence of chlorophyll in the A90 model. Unfortunately, we cannot know if this parameter, which is linked to the tropism, would have had an important role even in the B90 model. However, our final consideration is that the presence of FADs plays a role in both models, especially in the model in which their use is greater.

### Experimental session

Artificial reefs provide habitats mainly for the recruitment of juvenile fish, but they also increase the connectivity between adjacent habitats (facilitating the dispersion of fish larvae^40–42^). The experimental work highlighted different views of how the FAD system deployed at an increasing distance from the coast can influence species spatial patterns. The juveniles of *B*. *capriscus* and *T*. *trachurus* have distributions that correspond to areas with a higher presence of FADs far from the coast. The first species seems to be trapped in its route towards the coast, while the exclusively pelagic species *T*. *trachurus* is always associated with offshore FADs. *Caranx crysos* seems to show interest in the coastal approach. At the beginning of settlement the large sized and more developed fraction of this fish was already distributed in the most coastal areas, and so it was also there at the subsequent times. In contrast, in a previous study *Seriola dumerili* showed a greater abundance and sizes exclusively in FADs far from the coast meanwhile abundance and size decrease over time in the coastal FADs and lower depths^18^. Instead, in the coastal areas *S*. *dumerili* showed a pattern similar to *C*. *crysos* with the FADs near the coast facilitating the approach to the adult habitat^18^. Our results suggest that the offshore FAD system is more efficient in assisting *C*. *crysos* towards the coast comparatively to what was previously found for *S*. *dumerili*^18^. In the FAD system arranged in an offshore-inshore way, the young *S*. *dumerili* in the FADs far from the coast remained blocked, and the amberjacks in the most coastal FADs were facilitated in their transition to the nectobenthonic domain^18^. Otherwise, for *C*. *crysos* FADs act as a stepping stone route fish from offshore to the coast. Over time *C*. *crysos* shows a decrease of large-sized individuals in the FADs closer to the coast and shallow waters, similarly to what was previously reported for *S*. *dumerili*^18^. This has been suggested to be related to an increase in the ERI (effective range of influence^43^) linked to the growth of the species^18^ that would facilitate the fish’s association with the coastal FADs in shallow water, the encounter of bottom and the abandonment of the FADs.

Following the highlights of the experimental session, *C*. *crysos* had the greatest benefit from the presence of the FAD system according to an offshore-inshore gradient. However, in the MaxEnt model the type of disposition (i.e. lines versus random FAD versus no line) of the FADs did not have an important contribution other than their number. The FADs are probably utilized as a network rather than as lines on a wider scale. In this dense network *C*. *crysos* spends its period of coast approach growing^11^, feeding on zooplankton^10^ and limiting its predation by protecting itself through FADs. This protection occurs passively when the fishes are very small and live in proximity to the FADs^1,11^, but also when they are larger (from 10–15 cm) and use the FADs to protect themselves actively when they are attacked by predators^17^.

## Conclusion

This study shows that *C*. *crysos* has expanded its geographical distribution since 1990 from the southeast towards the central part of the Mediterranean Sea. The model for the period before 1990 (B90 model) explains that temperatures close to 20 °C and the tolerance to the variation of this variable (standard deviation) are associated with higher occurrence of this species. This is probably due to the fact that higher temperatures are found in southeast areas and even off Tunisia. In the B90 model, *C*. *crysos* also showed a low tolerance to salinity variations. This may justify its minor presence throughout the western Mediterranean area where salinity showed higher variation.

The model for the period after 1990 (A90 model) offers a different scenario because after chlorophyll, the number of FADs was the main variable explaining *C*. *crysos* spatial distribution. This model shows an affinity of *C*. *crysos* to meso/oligotrophic waters. These conditions occur in the coastal areas of the eastern Mediterranean and in the southern part of the central Mediterranean. In this last area *C*. *crysos* showed a greater increase of number of records compared to the eastern Mediterranean although the environmental conditions that drive the spatial pattern of the species are similar. The only difference between the two areas is the presence of FADs, higher in the in the southern part of the central Mediterranean. Therefore presence of FADs could be an important and discriminatory variable.

While the model highlighted that the FADs contributed to the spatial distribution of *C*. *crysos*, the experimental session explains how FADs benefit fishes. Indeed this system facilitates the dispersion of *C*. *crysos* to the coast as provides a shelter for juveniles approaching the coast. In addition the deployment modality of FADs (line vs. random) seems not to have a specific role in this process. The use of two other species for comparison suggested that *C*. *crysos* has greater and more specific advantages.

We highlighted for the first time how the extensive use of FADs may have played an important role in changes to the geographical distribution and abundance of a fish species on a large scale. Despite being an alien species, *C*. *crysos* is now very common in the southern Tyrrhenian area, to such an extent that it is also a constant resource for small-scale fisheries.

In the Mediterranean Sea variation in the sea temperature has affected the distribution of endemic fish through the expansion of thermophilic and alien fish species^8,44–46^. Our research suggests that fishing activities can facilitate this process, as has already been reported in other studies^44^. The extensive use of FADs in the Mediterranean Sea could play a role in altering fish biodiversity, and therefore fishery management plans should impose a strict control on their use. This should also take into account that there is no relationship between the number of FADs and their fishing yield^8^.

## Supplementary information

Appendix S1

Appendix S2 and S3

## Data Availability

The dataset generated and analyzed during the current study are available from the corresponding author on reasonable request.
