# Supplementary material for: Extensive use of Fish Aggregating Devices together with environmental change influenced the spatial distribution of a tropical affinity fish
Source: Sci Rep. 2019 Mar 20;9:4934. doi: 10.1038/s41598-019-41421-9 (PMC6426954; doi:10.1038/s41598-019-41421-9)
Supplement: Supplementary file 2 — Appendix S2 and S3 [file 41598_2019_41421_MOESM2_ESM.pdf]

Extensive use of Fish Aggregating Devices together with environmental change influenced the spatial distribution of a tropical affinity fish

Mauro Sinopoli*[1], Valentina Lauria[2], Germana Garofalo[2], Teresa Maggio[3], Tiziana Cillari[3]

[1] Stazione Zoologica Anton Dohrn Napoli, Villa Comunale, 80121, Napoli, Italy

[2]IRBIM - Istituto per le Risorse Biologiche e le Biotecnologie Marine, CNR - National Research Council, Via Luigi Vaccara 61, 91026 Mazara del Vallo (TP), Italy.

[3]Institute for Environmental Protection and Research (ISPRA), BIO-CIT. Lungomare Cristoforo Colombo n. 4521 (ex complesso Roosevelt) Località Addaura, 90149 Palermo ITALY.

[*]mauro.sinopoli@szn.it

Appendix S2 Results; *The maps of the six environmental variables*

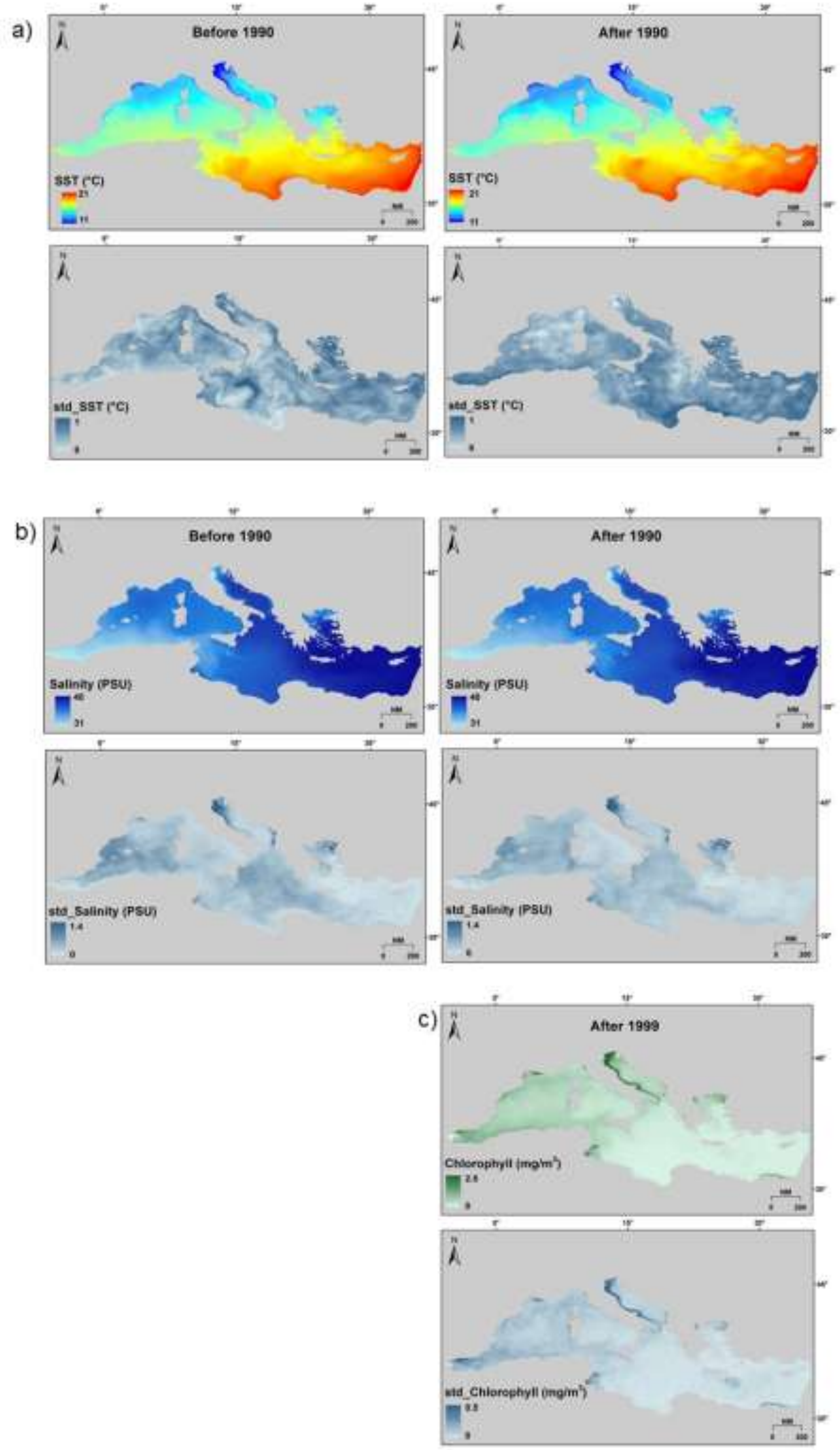

Figure S2 The spatial patterns of the environmental variables used to map the habitat models for the periods before and after 1990. These include (a) Sea Surface Temperature and its standard deviation; (b) Salinity and its standard deviation; (c) Chlorophyll and its standard deviation).

Extensive use of Fish Aggregating Devices together with environmental change influenced the spatial distribution of a tropical affinity fish

Mauro Sinopoli*[1], Valentina Lauria[2], Germana Garofalo[2], Teresa Maggio[3], Tiziana Cillari[3]

[1] Stazione Zoologica Anton Dohrn Napoli, Villa Comunale, 80121, Napoli, Italy

[2]IRBIM - Istituto per le Risorse Biologiche e le Biotecnologie Marine, CNR - National Research Council, Via Luigi Vaccara 61, 91026 Mazara del Vallo (TP), Italy.

[3]Institute for Environmental Protection and Research (ISPRA), BIO-CIT. Lungomare Cristoforo Colombo n. 4521 (ex complesso Roosevelt) Località Addaura, 90149 Palermo ITALY.

[*]mauro.sinopoli@szn.it

Appendix S3 Results; Caranx crysos distribution maps

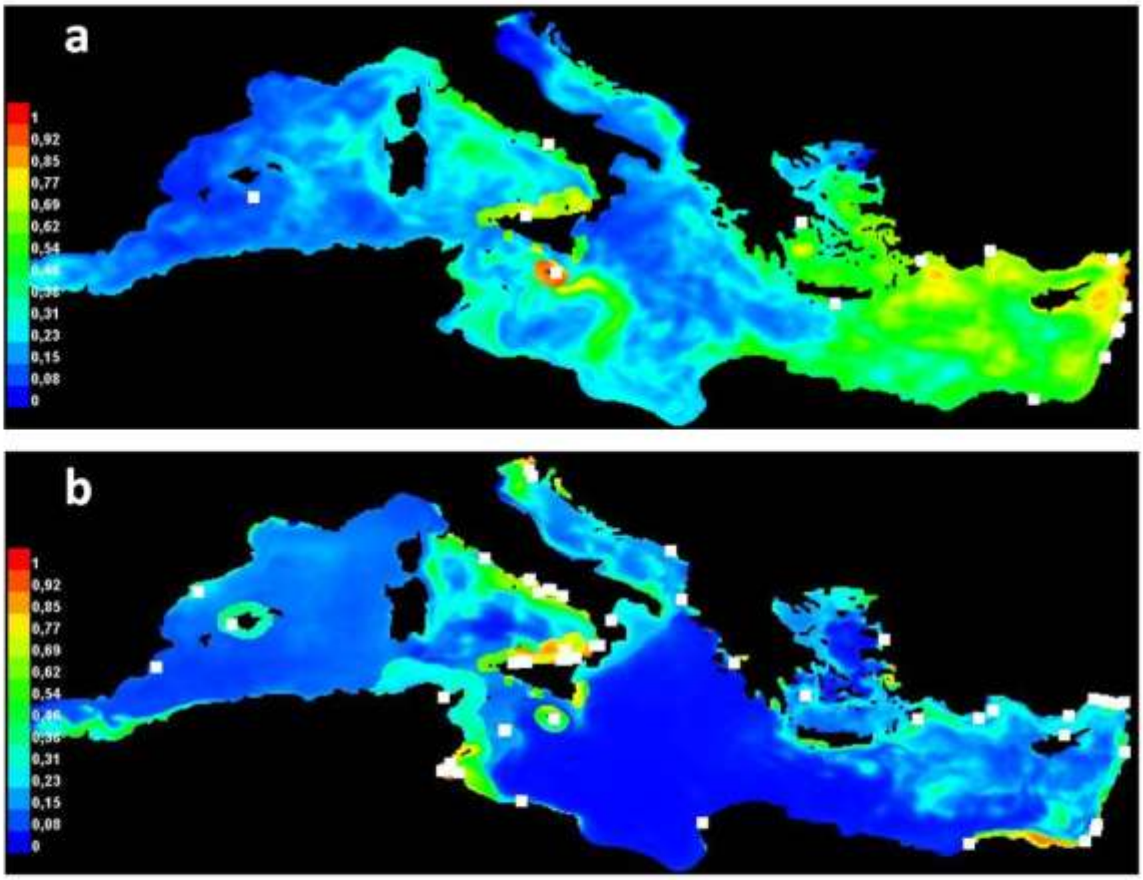

Figure S3 Predicted distribution from the MaxEnt representing the preferential habitat of *C. crysos* a) for the periods from1955 to 1990 (A90 model); b) for the periods from1990 to 2017 (P90 model). Colors indicate the likelihood of presence of the species for each cell, ranging from 0 to 1. White dots show the presence locations used for training.
